# Role of patients associations in connective tissue calcifiying diseases: a position statement from EuroSoftCalc.Net group

**DOI:** 10.1186/s13023-021-01703-w

**Published:** 2021-02-08

**Authors:** Pedro Valdivielso, Marta Jacinto, Guillemette Devernois, Jorge Laplana, Maria García-Fernández, Ludovic Martin

**Affiliations:** 1 Departamento de Medicina y Dermatología, School of Medicine, Spain and Instituto de Investigación Biomédica de Málaga (IBIMA), Facultad de Medicina, University of Málaga, Boulevard Luis Pasteur 32, 29071 Málaga, Spain; 2 Associação Pseudoxantoma Elástico Portugal, Lisbon, Portugal; 3 Association K20, Jouars-Pontchartrain, France; 4 Asociación Española de Afectados Por Pseudoxantoma Elástico, Malaga, Spain; 5 PXE Consultation Center, MAGEC Nord Reference Center for Rare Skin Diseases, Angers University Hospital, Angers, France

## Abstract

Patients have been showing a growing interest in taking active participation in decision making, and having the opportunity to drive clinical investigation. This is more common for patients who have a rare disease than for those with more prevalent diseases. The EuroSoftCalc.Net COST Action, a group of clinicians and researchers involved in the dystrophic calcification process held a meeting in which three representatives of patients’ associations, coming from Portugal, France and Spain, discussed the role of patients and their associations, namely in the Action, and also the main concerns in their countries. The disparities in health care between European Union countries with regard to connective tissue calcifying diseases, and the existing conflicts of interest, were a matter of debate during the meeting. As a consequence of the presentations and the debate that followed, it became clear that, despite their countries, the main concerns of the patients are identical, namely a lack of specific therapy and follow-up clinical guidelines, delays in the diagnosis, difficulties in getting members to enrol to associations, and/or difficulties with doctors’ explanations for the diseases. The attendees also agreed that EuroSoftCalc.Net group should help to set up new associations where no Patient Associations presently exist, and, furthermore, should release diagnosis and follow-up guidelines, especially helpful in countries, and/or for diseases, where no multidisciplinary consultations are available.

## Background

On the 15th of November 2018, a meeting funded by the COST Action 16-115, EuroSoftCalc.Net, held in Málaga, Spain, invited patients, clinicians and researchers to hold discussions regarding diseases caused by dystrophic calcification. The meeting was divided into two blocks. During the first block, clinicians and researchers presented up to date information on the diseases covered by the COST Action, mainly Pseudoxanthoma Elasticum (PXE) and Generalized Arterial Calcification of Infancy (GACI). The second block focused on the role of COST Action, patient advocacy groups, and the role of patients in the EMA committee to help leverage orphan drugs approval. This paper is dedicated primarily to the second block.

## Role of EuroSoftCalc.Net

COST (European Cooperation in Science and Technology) [1] is a time-honoured, transnational funding organization for interdisciplinary research networks in the European Union. Indeed, COST is, since 1971, committed to developing scientific collaboration all over the European continent and beyond, in all scientific fields. As of today, COST covers 38 countries, cooperating and international members, and it has funded dozens of Actions in Healthcare as well as various other areas. COST funds are limited to four-year periods and are dedicated to intensive networking and collaboration activities, such as workshops, conferences, working group meetings, time-limited training and/or scientific missions, and dissemination activities. Importantly, COST does not fund science directly but enables strong networks to be built, thus gaining the ability to apply for scientific programs, such as H2020.

People at COST noticed that “the nature of science has changed: it has become more interconnected, multidisciplinary, collaborative and data-intensive”, whilst also being “all about the people”. Therefore, COST Actions encourage open approaches that will mix the resources from both academia and industry, and, more specifically in the field of Health, ensure that the priceless input of patients and patient associations is taken into account. COST’s functioning is a “bottom-up” approach, further demonstrating that COST Actions promote the solving of complex medical/scientific issues through open yet tight field collaborations between researchers, physicians and patients.

Our COST Action (CA 16-115) is called EuroSoftCalc.Net (www.uantwerpen.be/en/projects/eurosoftcalcnet/). [2] and was launched in Brussels in April 2016. Its primary aim is to set up a transdisciplinary European network to share knowledge, expertise and training in pre-clinical and clinical research, as well as the management and treatment of people with connective tissue calcifying diseases (CTCDs) of rare and most often inherited origin. The conditions covered by this Action have been reviewed recently by our network [3]. In 2020, CTCDs remain a challenge for both researchers and people suffering with those rare conditions because their pathophysiology is still poorly understood, and guidelines for follow-up and care do not exist for most of the conditions. Working on CTCDs is also central for Public Health since the pathomechanisms that lead to ectopic calcifications in CTCDs are linked with many very common conditions, for instance, diabetes, atherosclerosis, chronic kidney diseases, rheumatologic conditions, as well as “normal” aging.

**Fig. 1:**
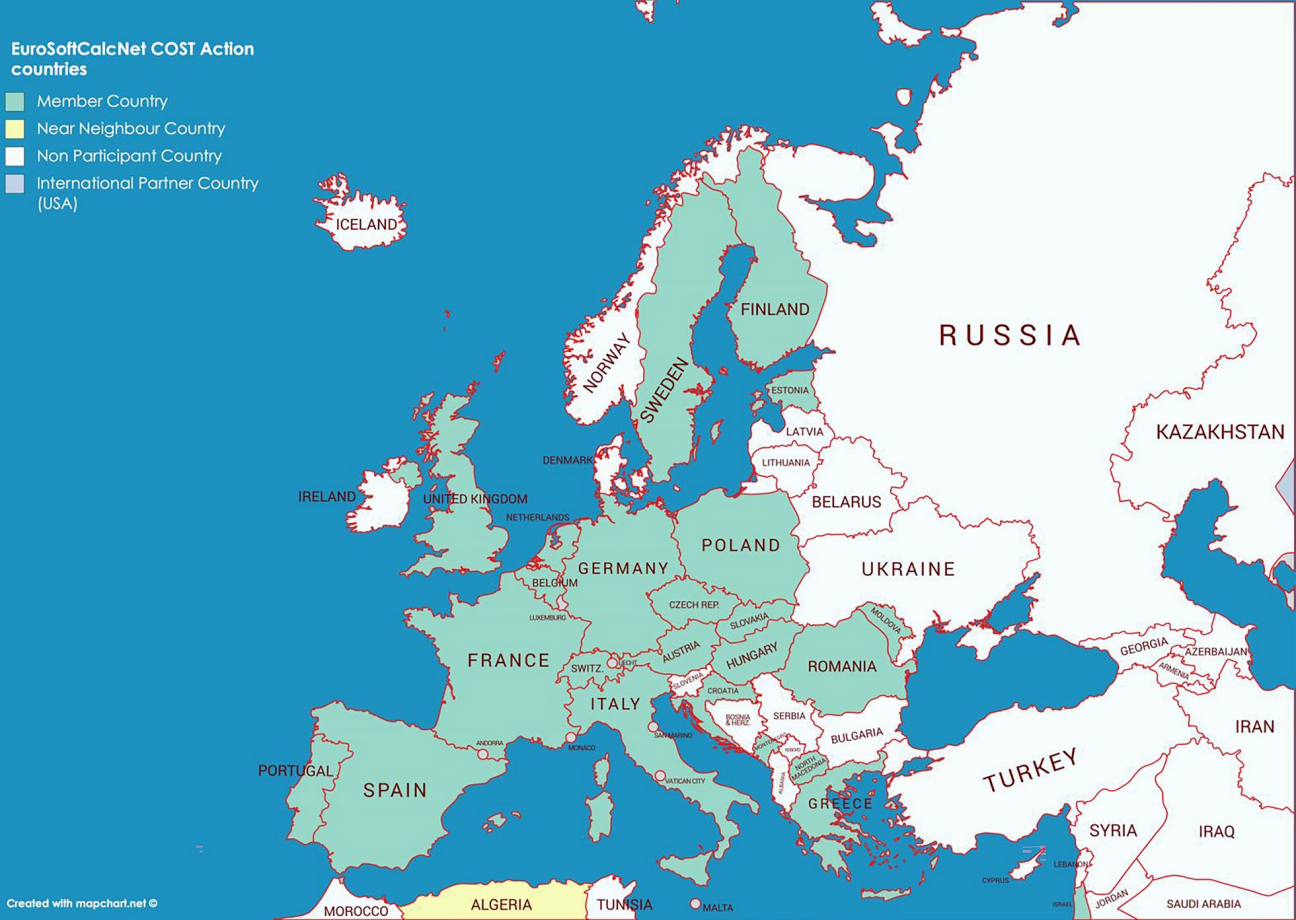
European countries contributing to the COST Action (CA 16-115), EuroSoft-Calc.Net

The objectives of the CA 16-115 have been listed in the so-called Memorandum of Understanding of the Action and may be found on the Action website [2]. Very briefly, our scientific objectives include: building a European cohort of more than 1000 CTCD people; building a multidisciplinary network gathering basic researchers, physicians (from many specialties), patients and Industry representatives to promote translational research on CTCDs; and paving the way for clinical therapeutic trials with innovative drugs that are able to counterbalance ectopic calcification. Figure 1 shows the countries that are members of EuroSoftCalc.Net and Table 1 shows Leadership, working groups and Management Committee Members. It is essential to note that our Action also involves patient associations. We aim to federate existing patient groups in several countries in order to promote the emergence of a powerful association which is visible at the European level. With close collaboration with the physicians, the patients are expected to be part of working groups that are dedicated to improving individual management all over Europe, maximizing inclusion in clinical trials and meeting with policy makers. According to recent research, there were only seven patient organizations in Europe dedicated to PXE (Germany, Spain, France, Portugal, Italy, UK and The Netherlands), and only one to GACI (Ireland). Unfortunately, not all of them are listed in the Orphanet website [4].

**Table 1:** Composition of COST Action CA 16,115 (EuroSoftCalcNet)

*Action leadership positions*		
Chair		Ludovic MARTIN (France)
Vice Chair		Tamas ARANYI (Hungary)
*Working Groups*		
WG# 1 (Building CTC cohorts)		Prof Olivier VANAKKER
WG# 2 (Translational research in CTC)		Dr Hervé KEMPF
WG# 3 (Clinical management of patients with CTC and impact studies)		Prof Frank RUTSCH
WG# 4 (Dissemination and patients’ activities)		Ms Sharon TERRY
WG# 5 (Scientific exchanges & training)		Prof Jessica BERTRAND
WG# 6 (Project coordinator)		Prof. Ludovic MARTIN
*Management Committee*		
Austria		Prof Gergely SZAKACS
Belgium		Prof Olivier VANAKKER,
		Prof Paul COUCKE
Croatia		Dr Dijana SONTACCHI,
		Dr Jurica PREDOVIĆ
Czech Republic		Dr Petra LISKOVA
Estonia		Prof Aare MÄRTSON
		Prof Sulev KÕKS
Finland		Dr Anamaria BALIC
France		Dr Hervé KEMPF
		Prof Georges LEFTHERIOTIS
Germany		Prof Frank RUTSCH
		Prof Jessica BERTRAND
Greece		Dr Maria YAVROPOULOU
Hungary		Dr Flóra SZERI
		Dr Tamas ARANYI
Israel		Dr Amiram ARIEL
		Prof Eli SPRECHER
Italy		Prof Daniela QUAGLINO
Moldova		Prof Ghenadie CUROCICHIN
Montenegro		Dr Antonia MATIJEVIĆ POT
		Dr Dusko MARKOVIC
Netherlands		Prof Leo JOOSTEN
North Macedonia		Dr Pavle ADAMOVSKI
Poland		Dr Krzysztof TOMASZEWSKI
Portugal		Ms Marta JACINTO
		Prof M. Leonor CANCELA
Romania		Dr Mihaela PERTEA
		Prof Anca CHIRIAC
Spain		Prof Pedro VALDIVIELSO
		Prof Victor SORRIBAS
Sweden		Prof Magnus BÄCK
Switzerland		Prof Alexander SO
		Prof Nathalie BUSSO
United Kingdom		Dr Vicky MACRAE
		Prof Catherine SHANAHAN

Table 2 summarizes the main characteristics of the diseases covered in the meeting [5–10]

**Table 2:** Main characteristic of CTCD

Condition	Orpha code	Gene	Prevalence	Symptoms
PXE	758	ABCC6	1–9/100,000	Loss of vision, skin papules, peripheral arterial disease
GACI	51,608	ENPP1	1/566,000	Arterial calcification, arterial stenosis, arterial hypertension, myocardial ischemia, premature death
PHP	97,953	PH1PA	1–9/1,000,000	Growth velocity decreasing progressively, Adult short stature, obesity, neurological impairment, advanced bone disease
CALJA	289,601	NT5E	< 1/1,000,000	Calcification of aneurysmally dilated arteries of lower extremity, intermittent claudication and limb ischemia

PXE, Pseudoxanthoma Elasticum; GACI, Generalized Arterial Calcification of Infancy; PHP, Pseudohypoparathydoidism; CALJA, CALcification of Joints and Arteries

The objective of this paper is to leverage the interventions, in the aforementioned meeting, of three representatives of National Patient Associations dedicated to CTCD, coming from France, Portugal and Spain, as well as to remark on some topics discussed following their speech, and to endorse the representatives’ feelings and considerations over the network.

## Patient Associations’ role

Patient Associations in CTCD, as in any other group of rare diseases, play an uttermost important role while dealing with patients, family members, caregivers and health professionals. Their role in informing and advising patients includes empowering the patients and family members, providing accurate and up to date information, and directing them to the right medical appointments. This role is probably the second most important task of these associations, for the most important one resides in bringing patients together so that they may share doubts, expectations and anxieties that occur following their diagnosis as being rare disease patients.

Some Patient Associations for rare diseases might also provide services to their members, complementing the ones offered by the National Health Systems (e.g. specific therapies, psychological or legal counselling). Furthermore, they often produce information for health professionals and they can also bridge patient-researcher interactions. Finding enough people so that researchers may work and collect funds are also two tasks that the associations perform.

Research in this sort of diseases is crucial because it will allow for better and faster diagnosis and, hopefully, lead to the existence of more orphan drugs. Patients should be involved directly in medical research, establishing direct contact with MD or PhD investigators, because this bi-directional engagement directly benefits the design, the participant recruitment to studies and the patients’ retention [11].

But that is just the tip of the iceberg, “Nothing about us without us” means that patients must be included and heard in every decision that concerns them, i.e. patients must be direct stakeholders in any commission, legislative matter, research program or RD protocol. This is beginning to happen more frequently, but it is still much rarer than it should be. Presently, even when being actively involved in projects or other initiatives, sometimes patients have a hard time getting through, being heard or having their contributions seriously considered, which means that they are not seen as real partners.

Patient advocacy, in turn, is likely to be achieved by raising awareness about the disease or group of diseases represented by the association.

The role of Patient Associations in rare CTCD’s is, in the end, broadly similar to the role of the remainder of rare diseases’ patient associations. This is mainly due to the fact that most types of concerns and difficulties that the patients face are transversal to rare diseases, for the main problems of a particular rare disease are similar to other rare diseases, for instance, delays in diagnosis; lack of registries or multidisciplinary consultations; patients not getting enough appointments for specific therapies; difficulty to, or the need to, provide fast and equal access to the existing orphan drugs to patients, just to name a few.

It is shown that Patient Associations may help to build up registries therefore alleviating some of these common problems. Registries may improve research in the area and, according to some authors, they also provide a historical cohort for one-armed clinical trials. Moreover, in the post-marketing phase of drug approval, a disease specific registry is likely to provide more relevant information than a product-specific registry [12].

This being said, it is only natural that Patient Associations gather at a national level in national umbrella institutions, the so-called National Alliances (NA), because 20, 100, or even 500 patients are unlikely to be able to make any government produce new laws or change their approach. This also enables best practices to be exchanged between diseases or groups of diseases, thus bringing a sense of togetherness that may also help with the creation of a RD registry at a national level. NA are the ones who will defend new policies and represent rare disease associations both nationally and in European or international organizations, unspecific to a disease.

The associations of some particular disease, or group of diseases, occasionally come together to create European or International Federations which will deal with the specific needs of that disease or group of diseases, therefore providing better representation of those particular associations, even helping to level up their actions.

At a transnational and European level, connecting both associations and their groups (Alliances and Federations), there is EURORDIS (Rare Diseases Europe). Besides many other tasks, this organization provides a great deal of information to their members and supports online help groups, thus helping particular associations to achieve their goals.

Table 3 summarizes the major roles of Patient Associations for both patients and doctors.

**Table 3:** Patient’s association roles

For patients	Informing
	Advising
	Empowering
	Giving accurate and up to date information
	Providing right medical appointments
	Psychological support
	Legal counselling
For Health Care Professionals	Information for better and faster diagnosis
	Recruiting patients for research
	Collecting funds for research
	Helping interactions between patients and doctors
	Building up Registries

## CTCD Patient Associations inside EuroSoftCalc.Net

The CTCD Patient Associations that are currently most involved in the EuroSoftCalc.Net COST Action are *Asociación Española de Afectados por Pseudoxantoma Elástico* (Spain) [13], *Association K20 (Pseudohypoparathyroidismo* [14] (France), and *Associação Pseudoxantoma Elástico Portugal* (Portugal) [15]. Coincidentally, both the associations dedicated to PXE are known as the expression 'PXE' followed by their 'country name' (PXE España and PXE Portugal).

For a Patient Association to be created, a considerable effort is needed from the initial members, and that goodwill could possibly run out over the months because those members are sometimes facing the animation of a non-­profit, "small business", alone. Challenges noticed by the associations are summarized in Table 4.

**Table 4:** Challenges in creating a Patient’s Association

Finding patients
Membership recruitment
Lack of interest in keeping affiliated
Scarce support from health system
Lack of reference units
Lack of funding

Improving diagnosis time and efficiency is something we are yet to achieve. In PXE, symptoms are very diverse and are dependent on the areas where the calcifications appear, and, in most cases, these calcifications won’t show up until they are clearly visible on the skin or in an ophthalmologic examination; diagnosis is, therefore, usually made by a dermatologist or an ophthalmologist. Notwithstanding, diagnosis often takes several years after the first signs appear regardless of the country the person lives in.

Similarly, Association K20 is also witnessing "late diagnosis". Indeed, they still hear, too often, that excessive delays occur between the appearance of the first symptoms and the diagnosis. This delay can cause desperation for patients and their families, generate errors, create a lack of treatment, cause accidents, deaths, as well as the birth of children disabled by these hereditary diseases. For the diseases represented by the Association K20, one of the causes of this inefficiency regarding diagnosis speed is the lack of a Reference Centre. Indeed, they feel there are not enough Reference Centres in France in relation to the number of rare diseases (363 in 2020). Moreover, some centres must manage neighbouring rare diseases because they are not listed in any centre at all. The Reference Centres then end up having too many diseases to manage and their work is slowed down. Even so, in Portugal, according to PXE Portugal, the situation regarding Reference Centres is much worse; only centres dealing with 6 rare disease areas were able to apply to be Reference Centres and, therefore, also able to join the European Reference Networks (ERN). Furthermore, no multidisciplinary consultations or Reference Centres are available for PXE in Portugal.

On the other hand, PXE patients in Spain have at their disposal a Reference PXE Unit in Hospital Virgen de la Victoria, Malaga, Spain. This Unit is composed of a multidisciplinary medical team consisting of internists, an ophthalmologist, a dermatologist, a gynaecologist and a clinical analyst, which care for all affected PXE patients.

Reaching this Unit from any place in Spain is, however, an arduous task, due to the lack of, or failures of, information channels available to all the doctors in order to refer patients who have already been diagnosed. PXE España stresses that it is necessary to integrate all of the Autonomic Registries for Rare Diseases into a National Registry to help health professionals nationwide. Similarly, they state that Reference European Networks are being created with the idea of sharing data and being a consultation database for health professionals, therefore helping them to find information for their patients’ treatments; patients are also involved in these networks through EURORDIS.

For PXE, having the national Reference Centres belonging to ERN may end up not meeting the Patient Associations’ expectations because, usually, the Reference Centre will adhere to the ERN of the leading doctor, which varies from country to country, so they may end up belonging to several ERN.

Association K20 states that, in France, in order to improve access to diagnosis, optimize patient care, or facilitate the path of the health professional, associations participate in various national projects in collaboration with their Reference Centres, such as emergency cards or PNDS (National Plan for Diagnostics and Care Protocols).

Emergency cards in France [16] are personal information cards that aim to improve the coordination of care for patients with rare diseases, especially in emergency situations. Distributed by specialized physicians, they indicate the symptoms that should be taken into account in the patient's assessment. These also summarize the actions to be taken or, on the contrary, to be avoided in an emergency situation. A similar card is currently issued in Portugal under the name Rare Disease Patient Card [17], however, it does not cover as many diseases as the French one and patients, especially the ones with diseases with no Orphan drugs available, state that it is not simple to obtain.

The French PNDS intends to make it possible to optimise and harmonise the management and monitoring of rare diseases throughout the country. These PNDS seek to establish clear diagnostic guidelines but, for the moment, few diseases benefit from a PNDS. The Reference Centres, even when supported by the associations, are usually perceived as progressing very slowly.

As mentioned by the K20 Association, Patient Associations frequently note that doctors are often very vague about the guidelines to be followed with regard to the diseases. This might be explained by the fact that these are rare diseases and that the medical profession is making slow progress in research and that there are still many unknowns. Nevertheless, for patients, hearing unclear answers and having to make decisions for themselves or their child is sometimes very difficult. Thus, the associations end up being a good relay for patients: the patient can express his fears and pose his or her questions. Without substituting the doctor, the association listens and can help in discernment.

As research is essential to understand the mechanisms of the disease and to find an effective treatment, PXE España, among others, believe that the most important goal is to support research. This research can be carried out with the help of public or private funds. In rare diseases, namely PXE, the resources are always limited because there are not many patients and the costs are very high. PXE España has collaborated actively in recruiting patients for an investigation on the role of 18F-NaF PET/CT scans assessing the severity of PXE in the skin and vessels, this was funded by ISCIII (Instituto de Salud Carlos III). Similarly, a clinical trial, which is ongoing and currently recruiting patients, at IBIMA (Instituto de Investigación Biomédica de Málaga—Biomedical Research Institute), was supported by a 20.000 euro donation from a private donor.

In PXE, as with many other rare diseases, there is currently neither cure nor treatment. Currently, the only thing that can be done is to deal with the consequences, whether it be intermittent claudication, retinal damage or something else. Also, all these diseases, being hereditary, require genetic testing and genetic counselling besides all the medical specialities that might be needed.

All the aforementioned associations have a dedicated website, are listed on the Orphanet website, and belong to the National Alliances that exist in their countries (FEDER—Spanish Federation of Rare Diseases in Spain, Alliance Maladies Rares in France and Aliança Portuguesa de Associações das Doenças Raras in Portugal). Additionally, PXE España, for example, also belongs to other federations for disabled patients.

PXE España feels that many of the symptoms produced by the PXE disease are incapacitating, thus forcing patients to ask for total or partial disability recognition from the administration. Social awareness and the ways that people ask for disability differ depending on the place a person lives or works at a European level. In Spain, the Spanish Non-Profit Public Entity ONCE (National Organization of the Blind in Spain) aims at improving life quality of blind, visually impaired and disabled people in Spain, including the adaptation of workplaces to support these individuals.

Patient Associations, besides listening to the patients, usually educate and give information to them and their relatives as well. People affected by rare diseases from the country where the Association exists, and other same language speaking countries, often contact the associations, usually by e-mail. For instance, in Spain, the association attends to the requests and, if the person asks for medical information, they refer the individual to the medical team, so that they can contact them directly.

Every two years PXE España organizes a national meeting between patients and doctors to allow everyone to have direct contact and to exchange concerns and projects.

Nevertheless, funding for the Patient Associations activities depends on members’ contributions, grants from public and private entities, and private donations meaning that resources are always limited, which considerably restricts the Patient Associations’ activities.

## Disparity in health care between EU countries with regard to CTCD disorders

CTCD disorders, and as many as 90% of other rare diseases, still lack an effective treatment [18]. However, inorganic pyrophosphate analogues, for example etidronate, have been used as therapy for PXE [19] and GACI [6]. For the case of PXE, anti-vascular endothelial growth factor (anti-VEGF) is currently used as an off-label drug to treat macular neovascularization to slow progression of retinal disease and visual impairment. Although no published data is available, a survey among members of EuroSoftCalc.Net showed that anti-VEGF drugs were reimbursed more frequently in western European countries than in eastern European countries. A similar trend is shown in general for orphan medicinal products. Back in 2015, 83 orphan medicinal drugs were available in Europe, special reimbursement being much higher in western European countries than in eastern European countries. Predictably, in absolute terms, higher income countries spent more on drugs [20]. Nevertheless, even in some high-income countries, not all the drugs are fully covered by national health insurance [21], demonstrating that the need for more awareness and investment across the continent is unequivocal.

## Conclusions

Patient Associations included in CTCD share almost the same concerns as many of the RD advocacy groups, namely a lack of specific treatments; delays in the diagnosis; difficulties regarding members enrolment; a lack of follow-up guidelines or doctors to explain the disease; and difficulties regarding the long-term follow up.

EurSoftCalc.Net members are convinced that Patient Associations should take an active and even major role on the COST Action over the coming years. In addition, the EuroSoftCalc.Net groups should promote the set-up of new associations in those numerous countries where no Patient Association presently exists. Moreover, diagnosis and follow-up guidelines should be produced for the CTCD included in the project, especially in order to warrant the correct guidance and management of patients living in countries where no multidisciplinary consultation is available, which, unfortunately, are often the same places where only less knowledgeable health professionals are available.

## Data Availability

Not applicable.
